# Tocolysis for inhibiting preterm birth in extremely preterm birth, multiple gestations and in growth-restricted fetuses: a systematic review and meta-analysis

**DOI:** 10.1186/s12978-015-0115-7

**Published:** 2016-01-14

**Authors:** Celine Miyazaki, Ralfh Garcia Moreno, Erika Ota, Toshiyuki Swa, Olufemi T. Oladapo, Rintaro Mori

**Affiliations:** 1 Department of Health Policy, National Center for Child Health and Development, Tokyo, Japan; 2 Graduate School of Human Sciences, Osaka University, Osaka, Japan; 3 Department of Reproductive Health and Research, World Health Organization, Geneva, Switzerland

**Keywords:** Extremely preterm birth, Growth-restricted babies, Meta-analysis, Multiple gestations, Non-randomized studies, Perinatal death, Prolongation of pregnancy, Randomized controlled trials, Tocolysis

## Abstract

This systematic review was to identify available evidence on the effectiveness of tocolysis in inhibiting preterm delivery for women with threatened extremely preterm birth, multiple gestations, and growth-restricted babies, and their infants’ outcomes. A comprehensive search using MEDLINE, Embase, the Cochrane Library, CINAHL, POPLINE and the WHO Global Health Library databases was conducted on 14 February 2014. For selection criteria, randomized controlled trials and non-randomized studies that compared tocolysis treatment to placebo or no treatment were considered. Selection of eligible studies, critical appraisal of the included studies, data collection, meta-analyses, and assessment of evidence quality were performed in accordance with the Cochrane Collaboration’s guidance and validated assessment criteria. The search identified seven studies for extremely preterm birth, in which three were randomized controlled trials (RCTs) and four were non-randomized studies (non-RCTs). There were no eligible studies identified for women with multiple pregnancy and growth-restricted fetuses. Meta-analyses indicated no significant difference was found for the relative effectiveness of tocolytics versus placebo for prolonging pregnancy in women with extremely preterm birth (RR 1.04, 95 % CI 0.83 to 1.31) or reducing the rate of perinatal deaths (RR 2.22, 95 % CI 0.26 to 19.24). In summary, there is no evidence to draw conclusions on the effectiveness of tocolytic therapy for women with threatened extremely preterm birth, multiple gestations, and growth-restricted babies.

**Electronic supplementary material:**

The online version of this article (doi:10.1186/s12978-015-0115-7) contains supplementary material, which is available to authorized users.

## Introduction

Preterm birth contributes significantly to the incidence of perinatal death, and other neonatal adverse outcomes [1, 2]. A systematic analysis estimated 14.9 million babies were born preterm, which constituting 11.1 % of all live births worldwide in the year 2010 [3]. Interventions provided to mothers during pregnancy have been perceived to reduce infant death and morbidity resulting from preterm birth [4]. In maternal preterm pregnancy management, tocolytics has been considered for women suspected with preterm labour at less than 37 weeks of pregnancy as an inhibiting-agent to suppress premature labour by inducing uterine quiescence or myometrial relaxation of the uterus [4, 5]. By delaying preterm delivery with the use of tocolytsis, gestational age could possibly be prolonged or alternative rescue treatments, such as corticosteroids for fetal lung development, could be administered during the extended interval to delivery [6, 7]. However, there is a concern about whether tocolytic treatments demonstrate the same efficacy or not for specified women with extremely preterm labour, multiple gestation or growth-restricted fetuses, and these specific conditions have not been fully evaluated independently.

A certain evidence-based report implied that tocolytic treatment were effective in prolonging pregnancy up to 7 days (single pregnancies) for women with preterm labour, including women at 24 to 27 weeks of pregnancy, but another report indicated that there was no significant difference in preterm birth for women with less than 28 weeks of gestation [8, 9]. Despite the fact that there are some contradictory evidence on effectiveness of tocolysis in delaying preterm birth or improving infant outcomes, guideline on preterm birth and labour have recommended the use of tocolysis for women diagnosed with spontaneous preterm labour under no severe complications, for example, placental abruption or intrauterine infection [4, 10]. To avoid increasing adverse effects from tocolytic treatment, contraindications of tocolysis have also been documented in the recommendations; however, some contraindications, such as extremely preterm birth, growth-restricted fetuses and multiple pregnancy, still are undetermined [11]. In order to diminish some of these variable and gaps, the effectiveness and safety of tocolytic treatment for women at high obstetric risk with either premature cervical dilation or unanticipated contractions that prompt imminent preterm delivery should be reviewed and addressed more specifically.

Thus, our objective was to systematically evaluate the effectiveness of tocolysis in inhibiting preterm birth among women with extremely preterm birth, multiple gestations and growth-restricted fetuses in the context of World Health Organization (WHO) guideline development.

## Review

### Methods

The reporting procedure for this systematic review was consistent to the checklist contained in the PRISMA statement for reporting systematic reviews and meta-analyses [12, 13].

#### Search strategy

A comprehensive search of MEDLINE, Embase, the Cochrane Library, CINAHL, POPLINE and the WHO Global Health Library databases was conducted on 14 February 2014 to identify studies that contained information about tocolysis for extremely preterm births, tocolysis in multiple pregnancies, and tocolysis in growth-restricted fetuses. No language restriction was imposed. The search was developed using related thesaurus terms and a wide range of free subject categories and/or keywords that met the objective of this review concept. Search terms included “tocolytic agents,” “prolonged pregnancy,” “premature,” “fetal development,” “maternal death,” “low birth weight,” “multiple pregnancy” and “restricted growth.” Further detail on the search strategy is provided in an additional file (see Additional file 1). All identified bibliography and reference studies were managed by using the EndNote® version 6 and Reference Manager® (Thomson Reuters, USA).

#### Selection and eligibility criteria

Titles and abstracts retrieved electronically were screened for potential eligibility by two researchers (CM and RMG) independently and consulted with technical advisory experts when necessary. Irrelevant or duplicated reports were removed and multiple reports for the same studies were link together as one study. Selected articles were then evaluated fully by their relevance in addressing one of the following health care questions for this systematic review: (1) Is tocolysis effective and safe for inhibiting extremely preterm birth?; (2) Is tocolysis effective and safe for inhibiting preterm birth in multiple pregnancy?; and (3) Is tocolysis effective and safe for inhibiting preterm birth in growth-restricted fetuses?. Disagreements on which studies should be included were resolved by discussions with other review authors or people in the advisory group. All potential full-text articles were examined by the compliance with the criteria as follow: (1) For extremely preterm birth, defined as birth before 28 weeks of gestation, pregnant women with threatened extreme premature labour (with uterine activity and/or cervical changes) were considered for inclusion and studies that recruited women of all gestational ages and provided a proper stratified analysis for extremely preterm births were considered eligible [3, 14]. (2) For multiple pregnancies, women who were carrying twins, triplets or more fetuses with high risk of preterm delivery were eligible for inclusion. The term “high risk of preterm delivery” encompasses preterm uterine activity and/or cervical changes. (3) Studies in which pregnant women with growth-restricted fetuses had high risk of preterm delivery and preterm uterine activity and/or cervical changes. The definition of growth-restricted fetuses was referred as the measurement by small for gestational age (SGA), which is defined by a birth weight less than the 10th percentile for gestational age, and is associated with preterm birth and other pregnancy complications related to preeclampsia or infections [15–17]. Studies involving the administrations of corticosteroids were considered eligible and studies that used any type of tocolysis (i.e. calcium channel-blockers, b-sympathomimetic, oxytocin inhibitors, among others) were included. Study designs, such as individual, cluster or quasi randomized controlled trials (RCTs), controlled before-after studies, prospective or retrospective cohorts with control groups, and, if necessary, case–control studies featuring one treatment group and a comparison group were considered for inclusion. Head-to-head studies were not included (e.g. atosiban vs. salbutamol).

#### Data collection and assessment of methodological quality

Two researchers independently conducted data collection from eligible studies by using a data extraction form, which was developed with the experts’ recommendation and in reference to the data collecting approach in *Cochrane Collaboration Handbook* [12]. Any discrepancy on specific data was resolved by consulting with a third researcher. To assess the internal quality of the studies, the Cochrane Collaboration’s tool for assessing risk of bias was used for each RCT study [12]. The risk of bias assessment tool was a domain-based evaluation: sequence generation, allocation concealment, blinding of participants and personnel, blinding of outcomes, incomplete outcome data, selective reporting and other bias. The judgment for risk of bias was made according to the criteria and the judgments were reported by assigning low risk, high risk and unclear risk to each domain. For non-randomized studies (non-RCTs), the Risk of Bias Assessment Tool for Nonrandomized Studies (RoBANS) was used [18]. The RoBANS tool composed of six domains: selection of participants, confounding variables, measurement of exposure, blinding of outcomes, incomplete outcome data and selective reporting. The assessment for risk of bias was made according to RoBANS criteria and the judgments were reported by assigning low risk, high risk and unclear risk to each domain.

#### Data synthesis

Data analyses were conducted by using a statistical software, Review Manager Version 5.3 (RevMan 5.3) [19]. Data from RCTs were assessed by meta-analyses, and data from non-RCTs (e.g. prospective and retrospective cohorts, case–control studies) were analyzed separately from RCTs and described narratively. Relative risk in terms of risk ratio (RR) was calculated from 2 by 2 table to measure the effect estimate for binary outcomes. All statistical analyses used a 95 % confidence interval and a *p*-value with a cut-off point of 0.05. Fixed-effect modeling was carried out to determine the effect estimates and a Chi^2^ statistic with a cut-off point of 0.10 was used to determine heterogeneity. The I^2^ statistic was used to assess the inconsistency among the studies, in which to detect the variability in the effect estimates due to heterogeneity. When the fixed-effects assumption could not provide the true effect of the intervention, a random-effects model was used. When treatment results showed statistical effectiveness, number needed to treat (NNT) was calculated from risk difference. To synthesize the data from the non-RCTs, the unadjusted relative risks were generated from 2 by 2 table, and when the data were considered to be appropriate, an estimate of the effect size was made. When unadjusted relative risk could not be obtained from the non-RCTs, their published results were presented accordingly and separately with either adjusted odds ratio (aOR), adjusted risk ratio (aRR) or adjusted hazard ratio (aHR) in this review.

#### Evidence grading

To rate quality of evidence, the Guideline Development Tool (Copyright © 2014, McMaster University and Evidence Prime Inc.) template was utilized. The assessment was made in compliance with the Grades of Recommendation, Assessment, Development and Evaluation Working Group (GRADE) guidelines, and the rating of evidence were determined by GRADE guidelines on study limitations criteria [20]. Quality rating was made for each outcome and was presented in four levels of quality recommended by The GRADE approach: high, then moderate, low and very low quality of body evidence. In terms of non-RCTs, quality of evidence rating began from low quality and could either be judged with very low, moderate or high quality of evidence as recommended in GRADE guidelines.

## Results

**Fig. 1:**
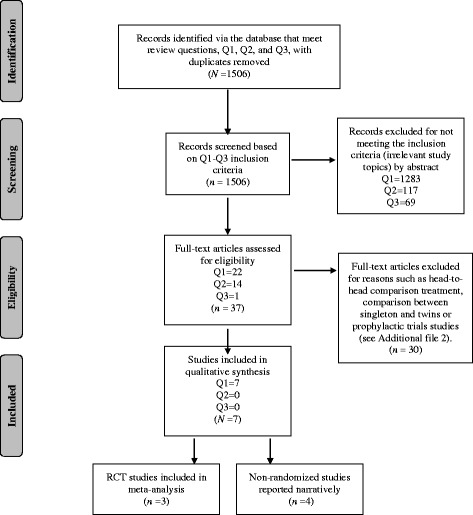
Flow diagram for review questions Q1-Q3

The comprehensive search in the bibliographic databases yielded a total of 1506 potential studies. The process of selecting eligible studies is presented in Fig. 1. The search for Q1, ‘Is tocolysis effective and safe for inhibiting preterm labour and delaying extremely preterm birth?’, retrieved 1305 titles and 22 studies were preselected after the initial screening process. Keywords linked to Q2, ‘Is tocolysis effective and safe for inhibiting preterm labour and delaying preterm birth in multiple gestations?’, retrieved 131 titles, and 14 studies were preselected. Search results linked to Q3, ‘Is tocolysis effective and safe for inhibiting preterm labour and delaying preterm birth growth-restricted fetuses?’, retrieved 70 titles, and only one study was preselected. Together, 37 preselected potential studies were examined thoroughly and 25 studies were excluded based on the criteria of this review. The excluded studies with reasons are presented in an additional file (see Additional file 2). After examining the full-text reports, seven studies were finalized for inclusion and analysis. The identified studies were conducted in the USA, Canada and Germany, and all the seven studies complied with the extremely preterm birth criteria. There were no eligible studies for multiple pregnancies, and growth-restricted fetuses. From the seven studies, two were RCTs [21, 22] and one was a prospective randomized trial by design [23]. The remaining four studies were retrospective cohorts by design [24–27]. The characteristics of the RCTs are presented in Table 1, and separately, the characteristics of the other four non-RCTs are presented in Table 2. The risk of bias assessment for individual studies resulted with two RCTs [21, 22] rated with unclear risk and one RCT [23] rated with high risk. For the non-RCTs, based on RoBANS assessment criteria, two retrospective studies [26, 27] were rated with unclear risk and the other two retrospective studies [24, 25] were rated with high risk of bias. The summary of risk of bias within studies is presented in an additional file (see Additional file 3).

**Table 1:** Characteristic of included studies (Randomized controlled trials)

Extremely preterm birth (less than 28 weeks of gestation)							
Study ID	Country	Study design	Sample size (groups)	Description of women/patients with preterm labour	Intervention (Description)	Comparison (Description)	Outcomes
Richter 2005 [23]	Germany	Prospective-RT	40 (*n* = 20 vs. *n* = 20)	Women, 31–42 years of age, between 18 to 24th week of gestation and with uterine contractions duration >30 s, rate ≥ 4/30 min. Cervical effacement >50 % and cervical dilatation of 0–3 cm (nulliparous), and 1–3 cm (primiparous and multiparous)	Atosiban	Placebo	Prolongation of pregnancy >48 h
					(Initial intravenous infusion of 6.75 mg of atosiban in 0.9 ml of sodium chloride, and followed by high dosage of infusion (300 lg/ min) for 3 h and then low dosage (100 lg/min) up to 45 h.)	(Intravenous infusion of saline solution)	Prolongation of pregnancy >7 days
Romero 2000 [21]	USA	RCT	501 (*n* = 250 vs. *n* = 251)	Women between gestational age of 20 weeks to 33 weeks, with intact membranes, cervical dilatation of 1 to ≤3 cm, preterm labor required the presence of ≥4 uterine contractions over 30 min, each lasting at least 40 s.	Atosiban	Placebo	Prolongation of pregnancy >24 h
			77 ^a^ (*n* = 43 vs. *n* = 34)		(Initial intravenous infusion of 6.75 mg of atosiban over 1 min and followed by an infusion of 300 μg/min of atosiban for 3 h, and then 100 μg/min for up to 45 h.)	(Matching placebo contained same formulation minus the 5 % mannitol solution of atosiban.)	Prolongation of pregnancy >48 h
							Prolongation of pregnancy >7 days
							Perinatal death
The Canadian PLIG 1992 [22]	Canada	RCT	708 (*n* = 352 vs. *n* = 356)	Women between gestational age of 20 to 35 weeks, with uterine contractions four per 20 min or six per 60 min or any uterine activity with ether rupture membranes or cervical dilatation by 2 cm or more.	Ritodrine	Placebo	Perinatal death
			151 ^a^ (*n* = 76 vs. *n* = 75)		(Intravenous infusion of ritodrine in 5 % dextrose at a rate of 0.35 mg/min until the cessation of uterine activity, the failure of therapy, or occurrence of impermissible maternal side effects	(Dextrose solution alone without ritodrine)	
Multiple gestations							
No report found for tocolytic treatment for imminent risk of preterm labor							
Growth-restricted fetuses							
No report found for tocolytic treatment for imminent risk of preterm labor							

^a^Subset of < 28 weeks of gestation sample size extracted from total participant in the study; (*n* = intervention group vs. *n* = comparison group); perspective-RT, prospective randomized trial

**Table 2:** A summary of effect size for tocolytic treatment versus placebo for extremely preterm birth outcomes in RCTs

Tocolysis compared to placebo in women with extremely preterm birth (RCTs)					
Patient or population: Women with extremely preterm birth					
Intervention: Tocolysis (atosiban and ritodrine)					
Comparison: Placebo					
Outcomes	Anticipated absolute effects^f^ (95 % CI)		Relative effect (95 % CI)	№ of participants (studies)	Quality of the evidence (GRADE)
	Risk with placebo	Risk with tocolysis			
Prolongation of pregnancy >24 h	Study population		RR 1.15 (0.81 to 1.63)	77 (1 RCT)	⨁⨁⨁ MODERATE ^a^
	59 per 100	68 per 100 (476 to 959)			
Prolongation of pregnancy >48 h	Study population		RR 1.40 (0.83 to 1.31)	117 (2 RCTs)	⨁ VERY LOW ^a,b^
	69 per 100	96 per 100 (57 to 90)			
Prolongation of pregnancy >7 days	Study population		RR 1.05 (0.75 to 1.48)	117 (2 RCTs)	⨁ VERY LOW ^a,b^
	65 per 100	68 per 100 (49 to 96)			
Perinatal death	Study population		RR 2.22 (0.26 to 19.24)	265 (2 RCTs)	⨁ VERY LOW ^c,d,e^
	17 per 100	37 per 100 (4 to 100)			
GRADE Working Group grades of evidence					
High quality: We are very confident that the true effect lies close to that of the estimate of the effect					
Moderate quality: We are moderately confident in the effect estimate: The true effect is likely to be close to the estimate of the effect, but there is a possibility that it is substantially different					
Low quality: Our confidence in the effect estimate is limited: The true effect may be substantially different from the estimate of the effect					
Very low quality: We have very little confidence in the effect estimate: The true effect is likely to be substantially different from the estimate of effect					
^a^Total number of cases less than 300					
^b^Allocation concealment not performed					
^c^One study with unclear randomization and one study without allocation concealment					
^d^Large heterogeneity (>60 %)					
^e^Small sample size (<300) and wide confidence interval					

*CI* Confidence interval; *RR* Risk ratio; *OR* Odds ratio

^f^The risk in the intervention group (and its 95 % confidence interval) is based on the assumed risk in the comparison group and the relative effect of the intervention (and its 95 % CI)

### Randomized controlled trials of tocolytic treatment for extremely preterm birth

Three RCTs for preterm birth including subset groups of women with extremely preterm birth were identified (Table 1). The three RCTs had a total of 1249 women and these women were with pregnancies from less than 28 weeks up to 35 weeks. All trials evaluated the use of tocolysis compared to placebo with a follow-up to delivery. Women with threatened extremely preterm labour with less than 28 weeks of gestation were recruited into the trials due to uterine contractions and/or cervical changes (e.g. dilatation or shortening). Two trials used atosiban in the treatment arm, and one trial used ritodrine. The subset results form the total population in regard to the effectiveness of tocolysis for extremely preterm birth (less than 28 weeks of gestation) were extracted from two trials for meta-analyses [21, 22].

#### Prolongation of pregnancy more than 24 and 48 h for extremely preterm birth (RCTs)

**Fig. 2:**
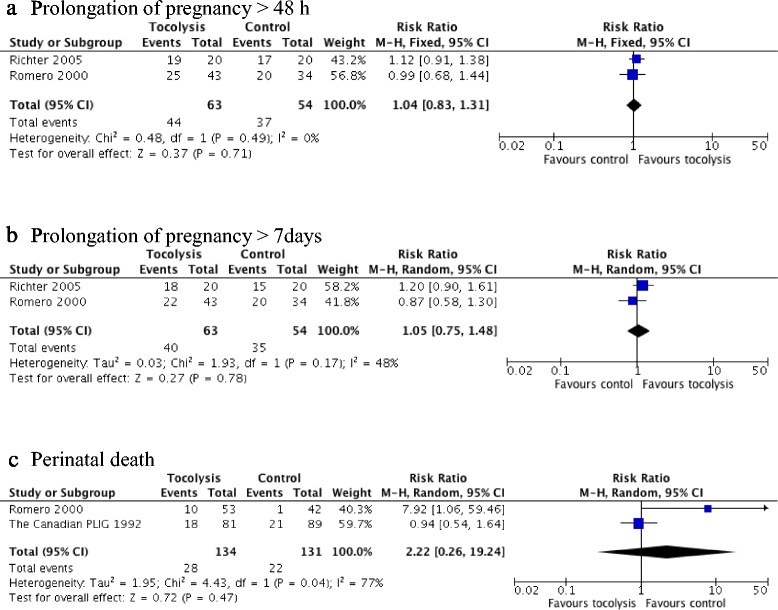
Forest plot for prolongation of delivery and perinatal death with RCTs that compared tocolytic treatment versus placebo for extremely preterm birth

Prolongation of pregnancy more than 24 h in women with extremely preterm birth was found in one trial [21] but there was no significant difference in the rate of prolonging the pregnancy between the tocolysis (atosiban) group and the placebo group (RR 1.15, 95 % CI 0.81 to 1.63, 77 women) (Table 2). For prolongation of pregnancy more than 48 h, there were two trials [21, 23] and the meta-analysis showed that there was no relative risk difference found between the tocolysis group and the placebo group (RR 1.04, 95 % CI 0.83 to 1.31, I^2^ = 0 %, 117 women). The effect estimate and the confidence interval crossed the line of no effect (Fig. 2a). There were no evident heterogeneity and inconsistency indicated among the studies.

#### Prolongation of pregnancy more than 7 days for extremely preterm birth (RCTs)

For prolongation of pregnancy more than 7 days, there were two trials [21, 23] and the pooled relative risk indicated no difference between the tocolysis group and the placebo group (RR 1.05, 95 % CI 0.75 to 1.48, I^2^ = 48 %, 117 women). The effect estimate and the confidence interval crossed the line of no effect (Fig. 2b). There was a substantial heterogeneity in this comparison and a moderate inconsistency across the studies.

#### Perinatal death in extremely preterm birth (RCTs)

Two RCTs [21, 22] reported on outcome of perinatal deaths and their combined perinatal deaths were 50 out of a total of 265 fetuses. The relative risk from these combined trials was RR 2.22, 95 % CI 0.26 to 19.24, I^2^ = 77 %, 265 fetuses (Fig. 2c). This estimate suggested that there was an increase risk of perinatal death with tocolytic treatment relative to placebo group, but the estimate showed statistical uncertainty due to wide confidence interval that crossed the line of no effect. A substantial heterogeneity and inconsistency were indicated among the studies.

#### Quality of the evidence from (RCTs) for extremely preterm birth

The quality of evidence for prolongation of pregnancy more than 24 h was rated to be moderate. However, the evidence for prolonging pregnancy more than 48 h or more than 7 days were rated very low due to small sample size, which contributed to imprecision, and one study [23] did not mention allocation concealment, which the limitation of the trial decrease the confidence in the estimated results. The quality of evidence for outcome of perinatal death was rated very low as well because of significant heterogeneity attributed by small sample size and the overall estimate had wide confidence interval, which indicated substantial uncertainly. The summary of quality of evidence is presented in GRADE tables (see Additional file 4).

### Non-randomized studies of tocolytic treatment for extremely preterm birth

Four non-RCTs of tocolytic treatment for women with extremely preterm labour were identified and they were all retrospective cohorts by design [24–27]. The identified non-RCTs evaluated tocolytic treatment versus to treatment, and two of the studies [24, 27] included cerclage with tocolysis treatment. Of the four studies, three studies [24, 25, 27] included information of 471 women who were at 14 to 23.9 weeks of pregnancy and one study [26] included only the information of 138 neonates of whom the mothers were at less than 29 weeks of pregnancy (Table 3). In regard to the types of tocolytics, three of the studies [24, 26, 27] evaluated indomethacin and one of the studies [25] evaluated any tocolytic medication (e.g. magnesium sulfate, indomethacin, nifedipine, used singly or in combination). The main outcomes and the unadjusted relative risks from these non-RCTs are presented in Table 4. The other outcomes, such as birth weight more than 1500 grams, intraventricular hemorrhage, necrotizing enterocolitis and patent ductus arteriosus, are presented in additional file (see Additional file 4).

**Table 3:** Characteristics of included studies (Non-randomized studies)

Extremely preterm birth (less than 28 weeks of gestation)							
Study ID	Country	Study design	Sample size	Description of women/patients with preterm labour	Intervention (description)	Comparison	Outcome
Berghella 2009 [27]	USA	Retrospective cohort (January 1998 - December 2005)	222 (*n* = 68 vs. *n* = 154)	Women between 14 and 25 week of gestation with suspected cervical dilation ≥1 cm.	Indomethancin plus some with cerclage	No treatment plus some with cerclage	Prolongation of pregnancy >28, 32, or 35 weeks
					(50 mg orally, followed by 25 mg orally every 6 h for a maximum of 48 h)		Perinatal death
							Birth weight >1500 grams
Cape 2010 [26]	USA	Retrospective cohort (2003–2008)	^a^138 neonates (*n* = 69 vs. *n* = 69)	All women less than 29 weeks of gestation with threaten with premature rupture of membranes.	Indomethancin	No treatment	Neonatal outcomes only including intraventricular hemorrhage, necrotizing enterocolitis, patent ductus arteriosus, and spontaneous intestinal perforations
Manuck 2012 [25]	USA	Retrospective cohort (January 2000 – June 2011)	148 (*n* = 84 vs. *n* = 64)	Women with a singleton non-anomalous fetus with spontaneous preterm labour and intact membranes, between 20–23.9 week of gestation, and with cervical dilation ≥ 1 cm and effaced > 50 %.	Tocolytic medication	No treatment	Prolongation of pregnancy >7 days
					(Database record of tocolytic treatment used i.e. magnesium sulfate, indomethacin or nifedipine, either used singly or in combination)		Perinatal death
Visintine 2008 [24]	USA	Retrospective cohort (1995–2006)	101 (*n* = 51 vs. *n* = 50)	Asymptomatic women followed from 14 weeks through 23 weeks 6 days gestation with a short cervical length, defined as <25 mm, placed with an ultrasound-indicated cerclage.	Indomethacin plus cerclage	Cerclage only	Prolongation of pregnancy >24, 32, or 35 weeks
					(50 mg initially orally or rectally, followed by 25 mg orally every 6 h for about 48 h.)		
Multiple gestations							
No report found for tocolytic treatment for imminent risk of preterm labor							
Growth-restricted fetuses							
No report found for tocolytic treatment for imminent risk of preterm labor							

^a^Sample size of infants exposed to *in utero* indomethancin within 4 weeks of delivery (*n* = intervenetion group vs. *n* = comparison group)

**Table 4:** A summary of results for tocolytic treatment versus no treatment for extremely preterm birth outcomes in non-RCTs

Tocolysis compared to no treatment for women with extremely preterm birth (non-RCTs)					
Patient or population: Women with extremely preterm birth					
Intervention: Tocolysis					
Comparison: no treatment					
Outcomes	Anticipated absolute effects ^h^(95 % CI)		Relative effect (95 % CI)	№ of participants (studies)	Quality of the evidence (GRADE)
	Risk with no treatment	Risk with tocolysis			
Prolongation of pregnancy >7 days	Study population		RR 2.13 (1.12 to 4.06)	148 (1 non-RCT)	⨁ VERY LOW ^a,b^
	16 per 100	33 per 100 (18 to 63)			
Prolongation of pregnancy >24 week	Study population		RR 0.91 (0.76 to 1.09)	101 (1 non-RCT)	⨁ VERY LOW ^c^
	86 per 100	78 per 100 (65 to 94)			
Prolongation of pregnancy >28 weeks	Study population		RR 0.91 (0.69 to 1.20)	222 (1 non-RCT)	⨁ VERY LOW ^d^
	55 per 100	50 per 100 (38 to 66)			
Prolongation of pregnancy >32 weeks	Study population		RR 0.94 (0.76 to 1.17)	323 (2 non-RCTs)	⨁ VERY LOW ^e^
	51 per 100	48 per 100 (39 to 60)			
Prolongation of pregnancy >35 weeks	Study population		RR 0.96 (0.75 to 1.23)	323 (2 non-RCTs)	⨁ VERY LOW ^e^
	43 per 100	41 per 100 (32 to 52)			
Neonatal survival	Study population		RR 1.12 (0.92 to 1.37)	222 (1 non-RCT)	⨁ VERY LOW ^d^
	62 per 100	69 per 100 (57 to 85)			
Perinatal death	Study population		RR 0.73 (0.55 to 0.95)	370 (2 non-RCT)	⨁ VERY LOW ^b,f^
	43 per 100	31 per 100 (24 to 41)			
GRADE Working Group grades of evidence					
High quality: We are very confident that the true effect lies close to that of the estimate of the effect					
Moderate quality: We are moderately confident in the effect estimate: The true effect is likely to be close to the estimate of the effect, but there is a possibility that it is substantially different					
Low quality: Our confidence in the effect estimate is limited: The true effect may be substantially different from the estimate of the effect					
Very low quality: We have very little confidence in the effect estimate: The true effect is likely to be substantially different from the estimate of effect					
^a^The information was from a study with high risk of confounding variables and unclear risk of outcome data reporting					
^b^A wide confidence interval without confounding variable adjusted					
^c^The information was from a study with high risk of selective reporting					
^d^The information was from a study with unclear risk of incomplete outcome data					
^e^The information was from two studies with unclear risk of incomplete outcome data in one study and high risk of selective reporting in another study					
^f^This information was from two studies with unclear risk of incomplete outcome data and on study with high risk of cofounding variables and unclear selective reporting					
^g^This information was from a study with unclear risk of selection of participants, measurement of exposure, incomplete data and selective reporting					

*CI* Confidence interval; *RR* Risk ratio; *OR* Odds ratio

^h^The risk in the intervention group (and its 95 % confidence interval) is based on the assumed risk in the comparison group and the relative effect of the intervention (and its 95 % CI)

#### Prolongation of pregnancy more than 7 days for extremely preterm birth (non-RCTs)

One non-RCTs [25] reported there was a relative different in rate between the tocolytic treatment group and the no treatment group for women remaining pregnant for 7 days or more after admission (RR 2.13, 95 % CI 1.12 to 4.06, 148 women; NNT = 6, 95 % CI 3.2 to 23.5) (Table 4).

#### Prolongation of pregnancy more than 24, 28, 32, or 35 weeks for extremely preterm birth (non-RCTs)

One study [24] reported that there was no difference in the rate of prolonging pregnancy for more than 24 weeks (RR 0.91, 95 % CI 0.76 to 1.09, 101 women); meanwhile, another study [27] also reported no significant difference was found in the rate of prolonging pregnancy for more than 28 weeks (RR 0.91, 95 % CI 0.69 to 1.20, 222 women) between the tocolytic treatment group and the no treatment group. Two non-RCTs reported on prolongation of pregnancy for more than 32 weeks: one study [27] showed that there was no difference found in the tocolytic treatment group relative to that in the no treatment group (RR 0.94, 95 % CI 0.68 to 1.30, 222 women) and another study [24] also showed a similar findings (RR 0.95, 95 % CI 0.73 to 1.24, 101 women). When the relative effect from these two non-RCTs [24, 27] were pooled, the overall estimate showed that there was no significant difference between the tocolysis (indomethacin) group and the no treatment group (RR 0.94, 95 % CI 0.76 to 1.17, I^2^ = 0 %, 323 women) (Table 4). Two non-RCTs [24, 27] reported on prolongation of pregnancy for more than 35 weeks. One study [27] showed that there was no significant difference found between the two comparison groups (RR 1.01, 95 % CI 0.68 to 1.48, 222 women), and similar result was reported in the other study [24] (RR 0.92, 95 % CI 0.68 to 1.24, 101 women). The overall effect estimate from these two non-RCTs [24, 27] indicated that there was no relative effect difference found between the tocolytic treatment group and the no treatment group (RR 0.96, 95 % CI 0.75 to 1.23, I^2^ = 0 %, 323 women) (Table 4).

#### Perinatal death in extremely preterm birth (non-RCTs)

Perinatal deaths were reported in two studies [25, 27] (Table 4). One study [27] showed that there was no significant difference found in the rate of neonatal survival (RR 1.12, 95 % CI 0.92 to 1.37, 222 fetuses) and the same indicated that there was no significant difference in the rate of perinatal deaths as well (RR 0.81, 95 % CI 0.54 to 1.21, 222 fetuses). The other study [25], in which several tocolytic were used, showed that there was a significance difference found in the rate of perinatal deaths and reported that the tocolysis group had 36.6 % of perinatal deaths as opposed to 62.5 % in the no treatment group (RR 0.65, 96%CI 0.45 to 0.94,148 fetuses; NNT = 6, 95 % CI 2.9 to 3.28, 148 fetuses). The overall effect estimate from the two non-RCTs [25, 27] showed a significant difference in reducing the rate of perinatal deaths (RR 0.73, 95 % CI 0.55 to 0.95, I^2^ = 0 %, 370 fetuses) and the pooled estimate implied that tocolytic treatment decreases the risk of perinatal deaths by 27 % (Table 4).

#### Quality of the evidence from (non-RCTs) for extremely preterm birth

The quality of evidence for prolongation of pregnancy for more than 7 days or 24, 28,32 and 35 weeks was rated very low because majority of the information were from studies with unclear risk of bias in the incomplete outcome data and high risk of bias in selective reporting and confounding variable. The quality of evidence for perinatal death was rated very low since the information was from two studies with unclear risk of bias for incomplete outcome data and selective reporting and one study was with high risk of bias for confounding variables (see Additional file 4).

### Randomized controlled trials and non-randomized studies of tocolytic treatment for multiple gestations

There were no eligible studies found for tocolytic treatment trials for inhibiting preterm birth for women with multiple pregnancies.

### Randomized controlled trials and non-randomized studies of tocolytic treatment for growth-restricted fetuses

There were no eligible studies found for tocolytic treatment for inhibiting preterm birth for women with growth-restricted fetuses, except a prophylactic controlled clinical trial for growth-restricted fetuses was identified [28].

## Discussion

Tocolytics for preterm labour has been documented in clinical recommendations and guidelines; however, they do not distinguished extremely preterm birth, multiple pregnancy and growth-restricted fetuses. This systematic review was to gather any available evidence of tocolytic treatment for specific group of women with imminent risk of preterm labour under extremely preterm, multiple pregnancy and growth-restricted fetuses conditions inclusively from RCTs and non-RCTs studies.

The meta-analysis data from the RCTs for women with extremely preterm labour showed that prolongation of pregnancy between tocolysis and placebo groups was largely uncertain since the prediction interval crossed the line of no effect. The overall effect estimate from these trials may not entirely represent only women with premature cervical dilation since one of the trial [23] included women with less then 20 weeks of gestation and the condition was connected with threaten miscarriage, preterm rupture of the membranes or infection after the trial enrollment. In addition, the subset data of women with less than 28 weeks of gestation was extracted from the larger preterm population, which could not be statistically powered for evaluating possible differential effects of the treatment due to small population size. Although the evidence was rated to be very low quality (one study without allocation and small population size), the analysis retained the fact that there was no definite evidence found for tocolytic treatment to be effective in prolonging pregnancy for women at less than 28 weeks of pregnancy and including those with high risk of miscarriage. This estimate was comparable to a systematic review, which also documented that uterine muscle relaxant drugs for women with threatened miscarriage for 170 women did not show to be effective in preventing preterm birth [29].

In regard to perinatal outcomes, perinatal death was primarily evaluated since neonatal and infant outcomes were too few as expected and were not distinguished clearly by gestational weeks or whether was from extremely preterm birth. The meta-analysis for perinatal death lacked the power to detect statistical significance due to substantial heterogeneity found among the studies and too few studies available to overcome such statistical requirement. The reason for such apparent heterogeneity could be the criteria difference in administrating alternative treatment across the studies, such as antenatal corticosteroids or glucocorticoid treatment. In the trial of using atosiban [21], 54 % of the treatment group received antenatal corticosteroids before 28 weeks of gestation after randomization in contrast with the ritodrine trial [22], where a proportion of women received full glucocorticoid treatment before randomization. Rescue treatments or additional treatments were the prominent cofounding factors, and future studies would need to consider covariance adjustment especially for imbalance baseline variable from the subset data. Apart from insufficient data to determine the true effect on perinatal death in extremely preterm birth, the administration of tocolytic treatments started on average at about 20 weeks of pregnancies estimated a trend towards no desirable benefit for reducing the risk of perinatal deaths. This observation was similar to the other systematic review reports on tocolysis in preterm birth management (less than 34 weeks of gestation) which also suggested no significant difference was found in perinatal death [8, 30].

In the non-RCTs, tocolytic treatment for women with threatened extreme preterm labour did not indicate effectiveness in prolonging pregnancy for more than 24, 28, 32 and 35 weeks based on each individual reporting. Although one of the non-RCT [25] showed a significant difference for prolonging pregnancy more than 7 days in women at 20 to 24 weeks of pregnancy, the average gestational age at delivery was 24.2 weeks in the tocolysis group and 23.2 weeks in the no treatment group. This report suggested that tocolytic treatment could not effectively inhibit extremely preterm birth or prolong the pregnancy to term delivery [5]. For outcome of perinatal death, two non-RCTs using indomethacin with cerclage treatment for extremely preterm birth reported different observations. One study [27] reported no significant difference in the rate of perinatal death under indomethacin, whereas the other study [25] implied that there was a significant difference in reducing the risk of perinatal death under several types of tocolytics (single or combined) used. The report on a lower rate of perinatal death found in tocolysis group was suggested by the short time gain from tocolytic treatments that increased the opportunity for providing antenatal corticosteroid treatment during the intervention. This particular study did not indicate confounding variables adjustment (e.g. women receiving one or women receiving more types of tocolytic were not specified) and the rate of neonatal death after receiving antenatal corticosteroids was not specified; therefore, caution is needed when interpreting some of these results.

Serious harm was not reported among the RCTs, as no maternal death occurred during the intervention period, though other adverse events, such as chest pain or tachycardia, were commonly reported. In the non-RCTs, harm assessment were not mentioned, but one study[26], in which newborns were exposed to indomethacin, reported a trend for patent ductus arteriosus surgical ligation (aHR 1.41, 95 % CI 0.93 to 2.14). For the most part, results from both RCTs and non-RCTs were reaching to parallel speculation in terms of the effectiveness of tocolysis for prolongation of pregnancy and reducing the rate of perinatal deaths under extremely preterm birth.

## Conclusions

There was no apparent effectiveness found for tocolytic treatment to inhibit preterm birth for women with extremely preterm condition or to reduce perinatal deaths, and there were no eligible studies found in regard to tocolytic treatment for multiple gestation and women with growth-restricted fetuses. Evidence from this review was not sufficient to provide specific recommendations for women with extremely preterm birth, and no conclusion could be drawn on the benefits or harms of tocolytic therapy in women carrying multiple pregnancies or growth-restricted fetuses at imminent risk of preterm birth. This review provided a trend of generic evidences that tocolysis is highly uncertain to be effective; therefore, practitioners and policy makers should reflect on its application to these populations.

## Additional files

Additional file 1: **Search strategy.** (DOCX 41 kb)

Additional file 2: **Excluded studies with reasons.** (DOCX 126 kb)

Additional file 3: **Risk of bias within studies.** (DOCX 188 kb)

Additional file 4: **GRADE tables.** (DOCX 134 kb)

## Abbreviations

aHR: adjusted hazard ratio
aOR: adjusted odd ratio
aRR: adjusted risk ratio
CI: confidence interval
GRADE: Grades of Recommendation, Assessment, Development and Evaluation Working Group
non-RCTS: non-randomized studies
NNT: Numbers needed to treat
OR: odd ratio
RCT: randomized controlled trial
RoBANS: risk of Bias Assessment Tool for Nonrandomized Studies
RR: Risk ratio
WHO: World Health Organization
