# Erratum to: ‘Tocolysis for inhibiting preterm birth in extremely preterm birth, multiple gestations and in growth-restricted fetuses: a systematic review and meta-analysis’

**DOI:** 10.1186/s12978-016-0127-y

**Published:** 2016-03-09

**Authors:** Celine Miyazaki, Ralf Moreno Garcia, Erika Ota, Toshiyuki Swa, Olufemi T. Oladapo, Rintaro Mori

**Affiliations:** Department of Health Policy, National Center for Child Health and Development, Tokyo, Japan; Graduate School of Human Sciences, Osaka University, Osaka, Japan; Department of Reproductive Health and Research, World Health Organization, Geneva, Switzerland

Unfortunately, the original version of this article [1] contained an error. The spelling of the author [Ralfh Moreno Garcia] name was incorrect. This has been corrected above and also included correctly below:

Ralf Moreno Garcia
