# Supplementary material for: Tocolysis for inhibiting preterm birth in extremely preterm birth, multiple gestations and in growth-restricted fetuses: a systematic review and meta-analysis
Source: Reprod Health. 2016 Jan 14;13:4. doi: 10.1186/s12978-015-0115-7 (PMC4712490; doi:10.1186/s12978-015-0115-7)
Supplement: Supplementary file 1 — Search strategy. (DOCX 41 kb) [file 12978_2015_115_MOESM1_ESM.docx]

**Additional file 1: Search strategy**

**1. MEDLINE**

RQ1. Is tocolysis effective and safe for inhibiting preterm labour and delaying preterm birth of growth restricted babies?*

RQ3. Is tocolysis effective and safe for inhibiting preterm labour and delaying preterm birth of growth restricted babies?*

February 14, 2014

| **ID** | **Search terms** |
| --- | --- |
| 1 | exp *Tocolytic Agents/ad, tu |
| 2 | exp *Tocolytic Agents/ and (ci or de of dt).fs. |
| 3 | *Tocolysis/ |
| 4 | exp Tocolytic Agents/ae, po, to |
| 5 | Tocolysis/ae |
| 6 | or/1-5 |
| 7 | exp *Obstetric Labor, Premature/pc |
| 8 | exp Fetal Development/ |
| 9 | exp Birth Weight/ |
| 10 | exp Infant, Low Birth Weight/ |
| 11 | or/7-10 |
| 12 | 6 and 11 |
| 13 | or/4-5 |
| 14 | exp Fetus/ |
| 15 | Obstetric Labor Complications/ |
| 16 | Pregnancy, Prolonged/ |
| 17 | exp Pregnancy Outcome/ |
| 18 | Fetal Death/ |
| 19 | Maternal Death/ |
| 20 | exp Infant, Newborn/ |
| 21 | Prenatal Exposure Delayed Effects/ |
| 22 | or/14-21 |
| 23 | 13 and 22 |
| 24 | or/12,23 |
| 25 | limit 26 to humans |
| 26 | limit 25 to (biography or case reports or comment or congresses or consensus development conference or consensus development conference, nih or editorial or guideline or historical article or interactive tutorial or interview or introductory journal article or lectures or news or newspaper article or overall or patient education handout or practice guideline or "review" or "scientific integrity review" or systematic reviews) |
| 27 | limit 26 to meta analysis |
| 28 | 26 not 27 |
| 29 | 25 not 28 |
| 30 | (tocoly* or Albuterol or Fenoterol or Hexoprenaline or Indomethacin or Isoxsuprine or Magnesium Sulfate or Nifedipine or Nylidrin or Ritodrine or Terbutaline).mp |
| 31 | ((((fetal or fetus or baby or babies or birth or infant* or neonate* or newborn* or labor or labour) adj2 (development or growth or matur* or weight or prematur* or preterm)) or (gestation* adj2 (age or period))) not ("patent ductus arteriosus" or rat* or animal*)).mp |
| 32 | 30 and 31 |
| 33 | MEDLINE.st. |
| 34 | 32 not 33 |
| 35 | (biograph* or case report* or comment or congress* or conference* or editor* or tutorial* or interview* or lecture* or news* or handout* or guideline* or (review* not (meta analys* or metaanalys*))).mp. |
| 36 | 34 not 35 |
| 37 | or/29,36 |
| 38 | *Ductus Arteriosus, Patent/ |
| 39 | 37 not 38 |

RQ2. Is tocolysis effective and safe for inhibiting preterm labour and delaying preterm birth in multiple gestation?*

February 14, 2014

| **ID** | **Search terms** |
| --- | --- |
| 1 | exp *Tocolytic Agents/ad, tu |
| 2 | exp *Tocolytic Agents/ and (ci or de of dt).fs. |
| 3 | *Tocolysis/ |
| 4 | exp Tocolytic Agents/ae, po, to |
| 5 | Tocolysis/ae |
| 6 | or/1-5 |
| 7 | exp Pregnancy, Multiple/ |
| 8 | exp Multiple Birth Offspring/ |
| 9 | or/7-8 |
| 10 | 6 and 9 |
| 11 | limit 10 to humans |
| 12 | limit 11 to (biography or case reports or comment or congresses or consensus development conference or consensus development conference, nih or editorial or guideline or historical article or interactive tutorial or interview or introductory journal article or lectures or news or newspaper article or overall or patient education handout or practice guideline or "review" or "scientific integrity review" or systematic reviews) |
| 13 | limit 12 to meta analysis |
| 14 | 12 not 13 |
| 15 | 11 not 14 |
| 16 | (tocoly* or Albuterol or Fenoterol or Hexoprenaline or Indomethacin or Isoxsuprine or Magnesium Sulfate or Nifedipine or Nylidrin or Ritodrine or Terbutaline).mp. |
| 17 | ((multiple adj3 (pregnanc* or birth* or offspring*)) or twin* or triplet* or quintuplet* or quadruplet*).mp. |
| 18 | 16 and 17 |
| 19 | MEDLINE.st. |
| 20 | 18 not 19 |
| 21 | (biograph* or case report* or comment or congress* or conference* or editor* or tutorial* or interview* or lecture* or news* or handout* or guideline* or (review* not (meta analys* or metaanalys*))).mp. |
| 22 | 20 not 21 |
| 23 | or/15,22 |

**2. EMBASE**

RQ1. Is tocolysis effective and safe for inhibiting preterm labour and delaying extremely preterm birth?*

RQ3. Is tocolysis effective and safe for inhibiting preterm labour and delaying preterm birth of growth restricted babies?*

February/13/2014

| **ID** | **Search terms** |
| --- | --- |
| #1 | 'uterus spasmolytic agent'/exp/mj/dd_do,dd_cm,dd_dt,dd_ad,dd_to,dd_ct,dd_it |
| #2 | 'uterus spasmolytic agent'/exp/dd_ae |
| #3 | 'tocolysis'/mj |
| #4 | 'tocolysis'/dd_ae |
| #5 | #1 OR #2 OR #3 OR #4 |
| #6 | 'immature and premature labor'/exp/mj |
| #7 | 'parameters concerning the fetus, newborn and pregnancy'/exp |
| #8 | 'fetus development'/exp |
| #9 | #6 OR #7 OR #8 |
| #10 | #5 AND #9 |
| #11 | #2 OR #4 |
| #12 | 'labor complication'/de |
| #13 | 'fetus'/de |
| #14 | 'newborn'/de |
| #15 | 'prenatal care'/exp |
| #16 | 'fetus death'/exp |
| #17 | 'prolonged pregnancy'/de |
| #18 | 'prenatal exposure'/de |
| #19 | #12 OR #13 OR #14 OR #15 OR #16 OR #17 OR #18 |
| #20 | #11 AND #19 |
| #21 | #10 OR #20 |
| #22 | #21 AND 'human'/de |
| #23 | #22 AND [embase]/lim NOT [medline]/lim |
| #24 | 'editorial'/de OR 'erratum'/exp OR 'note'/de OR 'review'/de |
| #25 | 'meta analysis'/exp |
| #26 | #24 NOT #25 |
| #27 | #23 NOT #26 |
| #28 | 'case report'/exp |
| #29 | #27 NOT #28 |
| #30 | 'patent ductus arteriosus'/exp/mj |
| #31 | #29 NOT #30 |

RQ2. Is tocolysis effective and safe for inhibiting preterm labour and delaying preterm birth in multiple gestation?*

February/13/2014

| **ID** | **Search terms** |
| --- | --- |
| #1 | 'uterus spasmolytic agent'/exp/mj/dd_do,dd_cm,dd_dt,dd_ad,dd_to,dd_ct,dd_it u |
| #2 | 'uterus spasmolytic agent'/exp/dd_ae |
| #3 | 'tocolysis'/mj |
| #4 | 'tocolysis'/dd_ae |
| #5 | #1 OR #2 OR #3 OR #4 |
| #6 | 'multiple pregnancy'/exp |
| #7 | #5 AND #6 |
| #8 | #7 AND 'human'/de |
| #9 | #8 AND [embase]/lim NOT [medline]/lim |
| #10 | 'editorial'/de OR 'erratum'/exp OR 'note'/de OR 'review'/de |
| #11 | 'meta analysis'/de |
| #12 | #10 NOT #11 |
| #13 | #9 NOT #12 |
| #14 | 'case report'/exp |
| #15 | #13 NOT #14 |

**3. Cochrane library**

RQ1. Is tocolysis effective and safe for inhibiting preterm labour and delaying extremely preterm birth?*

RQ2. Is tocolysis effective and safe for inhibiting preterm labour and delaying preterm birth in multiple gestation?*

RQ3. Is tocolysis effective and safe for inhibiting preterm labour and delaying preterm birth of growth restricted babies?*

February14, 2014

| **ID** | **Search terms** |
| --- | --- |
| #1 | MeSH descriptor: [Tocolytic Agents] explode all trees |
| #2 | MeSH descriptor: [Tocolysis] explode all trees |
| #3 | Tocoly* or Magnesium Sulfate or Ritodrine or Terbutaline or spasmoly* |
| #4 | #1 or #2 or #3 |
| #5 | MeSH descriptor: [Obstetric Labor, Premature] explode all trees |
| #6 | prematur* or immatur* or matur* |
| #7 | MeSH descriptor: [Fetal Development] explode all trees |
| #8 | MeSH descriptor: [Birth Weight] explode all trees |
| #9 | (fetal or fetus or birth) near weight |
| #10 | MeSH descriptor: [Fetus] explode all trees |
| #11 | MeSH descriptor: [Obstetric Labor Complications] explode all trees |
| #12 | MeSH descriptor: [Pregnancy, Prolonged] explode all trees |
| #13 | MeSH descriptor: [Pregnancy Outcome] explode all trees |
| #14 | MeSH descriptor: [Fetal Death] explode all trees |
| #15 | MeSH descriptor: [Maternal Death] explode all trees |
| #16 | MeSH descriptor: [Infant, Newborn] explode all trees |
| #17 | MeSH descriptor: [Prenatal Exposure Delayed Effects] explode all trees |
| #18 | "fetus mortality":kw or "fetus outcome":kw or "fetus risk":kw or "fetus mortality":kw or "fetus weight" or "gestational age":kw or "live birth" or "pregnancy outcome":kw or "prenatal mortality":kw or fetus:kw or newborn:kw or "labor inhibition":kw or "prenatal care":kw or "perinatal mortality":kw or "prenatal development" or "fetus development" or "prenatal exposure":kw |
| #19 | #5 or #6 or #7 or #8 or #9 or #10 or #11 or #12 or #13 or #14 or #15 or #16 or #17 or #18 |
| #20 | #4 and #5 |
| #21 | handsrch |
| #22 | #20 and #21 |
| #23 | restrict* near growth |
| #24 | #20 and #23 |

RQ2. Is tocolysis effective and safe for inhibiting preterm labour and delaying preterm birth in multiple gestation?*

February 14, 2014

| **ID** | **Search terms** |
| --- | --- |
| #1 | MeSH descriptor: [Tocolytic Agents] explode all trees |
| #2 | MeSH descriptor: [Tocolysis] explode all trees |
| #3 | Tocoly* or Magnesium Sulfate or Ritodrine or Terbutaline or spasmoly* |
| #4 | #1 or #2 or #3 |
| #5 | MeSH descriptor: [Pregnancy, Multiple] explode all trees |
| #6 | MeSH descriptor: [Multiple Birth Offspring] explode all trees |
| #7 | "multiple pregnancy":kw |
| #8 | (multiple near (pregnancy or birth)) or twin* |
| #9 | #5 or #6 or #7 or #8 |
| #10 | #4 and #9 |
| #11 | handsrch |
| #12 | #10 and #11 |

**4. CINAHL**

RQ1. Is tocolysis effective and safe for inhibiting preterm labour and delaying extremely preterm birth?*

RQ3. Is tocolysis effective and safe for inhibiting preterm labour and delaying preterm birth of growth restricted babies?

February 14, 2014

| **ID** | **Search terms** |
| --- | --- |
| S1 | (MM "Tocolytic Agents+/AD/DE/TU") |
| S2 | (MH "Tocolytic Agents+/PO/AE") |
| S3 | S1 or S2 |
| S4 | (MH "Labor, Premature") |
| S5 | (MH "Fetal Development+") |
| S6 | (MH "Birth Weight") |
| S7 | (MH "Infant, Low Birth Weight+") |
| S8 | S4 or S5 or S6 or S7 |
| S9 | S3 and S8 |
| S10 | (MH "Fetus+") |
| S11 | (MH "Labor Complications") |
| S12 | (MH "Pregnancy, Prolonged") |
| S13 | (MH "Pregnancy Outcomes") |
| S14 | (MH "Perinatal Death") |
| S15 | (MH "Maternal Mortality") |
| S16 | (MH "Infant, Newborn+") |
| S17 | (MH "Prenatal Exposure Delayed Effects") |
| S18 | S10 or S11 or S12 or S13 or S14 or S15 or S16 or S17 |
| S19 | S2 and S18 |
| S20 | S9 or S19 |
| S21 | S20 Limiters - Human |
| S22 | S21 Limiters - Research Article; Exclude MEDLINE records |

RQ2. Is tocolysis effective and safe for inhibiting preterm labour and delaying preterm birth in multiple gestation?*

February 14, 2014

| **ID** | **Search terms** |
| --- | --- |
| S1 | (MM "Tocolytic Agents+/AD/DE/TU") |
| S2 | (MH "Tocolytic Agents+/PO/AE") |
| S3 | S1 or S2 |
| S4 | (MH "Pregnancy, Multiple+") |
| S5 | (MH "Multiple Offspring+") |
| S6 | S4 or S5 |
| S7 | S3 and S6 |
| S8 | S7 Limiters - Human |
| S9 | S8 Limiters - Research Article; Exclude MEDLINE records |

**5. POPLINE**

RQ1. Is tocolysis effective and safe for inhibiting preterm labour and delaying extremely preterm birth?*

February 14, 2014

| **ID** | **Search terms** |
| --- | --- |
| 1 | (tocoly* OR indomethacin* OR "Magnesium Sulfate" OR Ritodrine OR Terbutaline OR spasmoly*) AND (labor OR labour OR premature OR immature OR matur*) NOT (restrict* AND growth) |

RQ2. Is tocolysis effective and safe for inhibiting preterm labour and delaying preterm birth in multiple gestation?*

February 14, 2014

| **ID** | **Search terms** |
| --- | --- |
| 1 | (tocoly* OR indomethacin* OR "Magnesium Sulfate" OR Ritodrine OR Terbutaline OR spasmoly*) AND (multiple OR twin OR triplet) |

RQ3. Is tocolysis effective and safe for inhibiting preterm labour and delaying preterm birth of extremely preterm pregnancies?

February 14, 2014

| **ID** | **Search terms** |
| --- | --- |
| 1 | (tocoly* OR indomethacin* OR "Magnesium Sulfate" OR Ritodrine OR Terbutaline OR spasmoly*) AND restrict* AND growth |

**6. WHO Global Health Library**

RQ1. Is tocolysis effective and safe for inhibiting preterm labour and delaying extremely preterm birth?*

February 14, 2014

| **ID** | **Search terms** |
| --- | --- |
| 1 | (tocoly* OR indomethacin* OR "Magnesium Sulfate" OR Ritodrine OR Terbutaline OR spasmoly*) AND (labor OR labour OR premature OR immature OR matur*) NOT (restrict* AND growth) |

RQ2. Is tocolysis effective and safe for inhibiting preterm labour and delaying preterm birth in multiple gestation?*

February 14, 2014

| **ID** | **Search terms** |
| --- | --- |
| 1 | (tocoly* OR indomethacin* OR "Magnesium Sulfate" OR Ritodrine OR Terbutaline OR spasmoly*) AND (multiple OR twin OR triplet) |

RQ3. Is tocolysis effective and safe for inhibiting preterm labour and delaying preterm birth of extremely preterm pregnancies?

February 14, 2014

| **ID** | **Search terms** |
| --- | --- |
| 1 | (tocoly* OR indomethacin* OR "Magnesium Sulfate" OR Ritodrine OR Terbutaline OR spasmoly*) AND restrict* AND growth |
