# Supplementary material for: Tocolysis for inhibiting preterm birth in extremely preterm birth, multiple gestations and in growth-restricted fetuses: a systematic review and meta-analysis
Source: Reprod Health. 2016 Jan 14;13:4. doi: 10.1186/s12978-015-0115-7 (PMC4712490; doi:10.1186/s12978-015-0115-7)
Supplement: Supplementary file 2 — Excluded studies with reasons. (DOCX 126 kb) [file 12978_2015_115_MOESM2_ESM.docx]

**Additional file 2: Excluded studies with reasons**

| **a. Extremely preterm birth** | | |
| --- | --- | --- |
| **No.** | **Study** | **Reason(s) for exclusion** |
| 1 | Al-Omari, W. R., H. B. Al-Shammaa, E. M. Al-Tikriti and K. W. Ahmed (2006). “Atosiban and nifedipine in acute tocolysis: a comparative study.” European Journal of Obstetrics, Gynecology, & Reproductive Biology **128**(1-2): 129-134. | Head-to-head treatment: atosiban vs. nifedipine. |
| 2 | Bomba-Opon, D. A., K. Kosinska-Kaczynska, P. Kosinski, P. Wegrzyn, B. Kaczynski and M. Wielgos (2012). “Vaginal progesterone after tocolytic therapy in threatened preterm labor.” Journal of Maternal-Fetal & Neonatal Medicine **25**(7): 1156-1159. | Head-to-head treatment: Progesterone vs. other tocolytics. Moreover, the study did not provide specific figures for pregnancies before 28 weeks. |
| 3 | Cabar, F. R., R. E. Bittar, C. M. Gomes and M. Zugaib (2008). “O atosibano como agente tocolítico: uma nova proposta de esquema terapêutico Atosiban as a tocolytic agent: a new proposal of a therapeutic approach.” Rev. Bras. Ginecol. Obstet **30**(2): 87-92. | Head-to-head treatment: atosiban vs. terbutaline. |
| 4 | Cox, S. M., M. L. Sherman and K. J. Leveno (1990). “Randomized investigation of magnesium sulfate for prevention of preterm birth.” American Journal of Obstetrics & Gynecology **163**(3): 767-772. | The results for pregnancies under 28 weeks have as denominator the total population (i.e. pregnancies <37 weeks), thus the study did not conduct real stratification. |
| 5 | European Atosiban Study, G. (2001). “The oxytocin antagonist atosiban versus the beta-agonist terbutaline in the treatment of preterm labor. A randomized, double-blind, controlled study.” Acta Obstetricia et Gynecologica Scandinavica 80(5): 413-422. | Head-to-head treatment: atosiban vs. terbutaline |
| 6 | French/Australian Atosiban Investigators, G. (2001). “Treatment of preterm labor with the oxytocin antagonist atosiban: a double-blind, randomized, controlled comparison with salbutamol.” European Journal of Obstetrics, Gynecology, & Reproductive Biology **98**(2): 177-185. | Head-to-head treatment: atosiban vs. salbutamol |
| 7 | Gummerus, M. (1981). “The management of premature labor with salbutamol.” Acta Obstetricia et Gynecologica Scandinavica **60**(4): 375-377. | Head-to-head treatment: salbutamol vs. fenoterol. |
| 8 | Hehir, M. P., H. D. O’Connor, E. M. Kent, M. S. Robson, D. P. Keane, M. P. Geary and F. D. Malone (2012). “Early and late preterm delivery rates - a comparison of differing tocolytic policies in a single urban population.” Journal of Maternal-Fetal & Neonatal Medicine **25**(11): 2234-2236 | The results for pregnancies under 28 weeks have as denominator the total population (i.e. pregnancies <37 weeks), thus the study did not conduct real stratification. |
| 9 | Husslein, P., L. Cabero Roura, J. W. Dudenhausen, H. Helmer, R. Frydman, N. Rizzo and D. Schneider (2007). “Atosiban versus usual care for the management of preterm labor.” Journal of Perinatal Medicine **35**(4): 305-313. | Head-to-head treatment: atosiban vs. other tocolytics (B-mimetics, Calcium channel blockers, Magnesium sulphate). |
| 10 | Katz, Z., M. Lancet, M. Yemini, B. M. Mogilner, A. Feigl and H. Ben Hur (1983). “Treatment of premature labor contractions with combined ritodrine and indomethacine.” International Journal of Gynaecology & Obstetrics **21**(4): 337-342. | Head-to-head treatment: ritodrine +indomethacine vs. ritodrine. |
| 11 | Locci, M., G. Nazzaro, A. Merenda, M. L. Pisaturo, P. Laviscio, R. Poppiti, M. Miranda, A. Stile and G. De Placido (2006). “Atosiban vs ritodrine used prophylactically with cerclage in ICSI pregnancies to prevent pre-term birth in women identified as being at high risk on the basis of transvaginal ultrasound scan.” Journal of Obstetrics & Gynaecology **26**(5): 396-401. | Head-to-head treatment: atosiban vs ritodrine. |
| 12 | Nonnenmacher, A., H. Hopp and J. Dudenhausen (2009). “[Effectiveness and safety of atosiban vs. pulsatile administration of fenoterol in the treatment of preterm labour].” Zeitschrift fur Geburtshilfe und Neonatologie **213**(5): 201-206. | Head-to-head treatment: atosiban vs. fenoterol. |
| 13 | Goodwin TM, Paul R, Silver H, Spellacy W, Parsons M, Chez R, et al. (1994). “The effect of the oxytocin antagonist atosiban on preterm uterine activity in the human.” Am J Obstet Gynecol.;**170**(2):474-8. | Unable to identify less then 28 weeks’ gestation data from pooled assessment between 20 to 36 weeks gestation. |
| 14 | Salim, R., G. Garmi, Z. Nachum, N. Zafran, S. Baram and E. Shalev (2012). “Nifedipine compared with atosiban for treating preterm labor: a randomized controlled trial.” Obstetrics & Gynecology **120**(6): 1323-1331. | Head-to-head treatment: nidedipine vs. atosiban. |
| 15 | Shim, J. Y., Y. W. Park, B. H. Yoon, Y. K. Cho and J. H. Yang (2006). “Multicentre, parallel group, randomised, single-blind study of the safety and efficacy of atosiban versus ritodrine in the treatment of acute preterm labour in Korean women.” BJOG: An International Journal of Obstetrics and Gynaecology **113**(11): 1228-1234. | Head-to-head treatment: atosiban vs. ritodrine |
| **b. Multiple gestations** | | |
| **No.** | **Study** | **Reason(s) for exclusion** |
| 1 | Al Nuaim, L. A. “Management of triplet pregnancy.” KMJ - Kuwait Medical Journal 33(3): 220-225.  Reason(s): | Although this study implemented tocolysis, the treatment was given only to those women who presented uterine contractions; the group of women who did not have uterine contractions was not given any medication. Thus, the two groups were not comparable since the risk factors and reasons for providing tocolysis were different. The study did not adjust for confounders, so any conclusion extracted from this comparison would be highly biased/misleading. |
| 2 | de la Torre, L., N. B. Istwan, C. Desch, D. J. Rhea, L. Roca, G. J. Stanziano and V. H. Gonzalez-Quintero (2008). “Management of recurrent preterm labor in twin gestations with nifedipine tocolysis.” American Journal of Perinatology 25(9): 555-560. | Head-to-head treatment: nifedipine vs. terbutaline. |
| 3 | Derbent, A., S. Simavli, I. I. Gumus, M. M. Tatli and N. O. Turhan (2011). “Nifedipine for the treatment of preterm labor in twin and singleton pregnancies.” Archives of Gynecology & Obstetrics 284(4): 821-826. | Comparison between singleton vs. twin pregnancies in which both groups were given nifedipine. |
| 4 | French/Australian Atosiban Investigators, G. (2001). “Treatment of preterm labor with the oxytocin antagonist atosiban: a double-blind, randomized, controlled comparison with salbutamol.” European Journal of Obstetrics, Gynecology, & Reproductive Biology 98(2): 177-185. | Head-to-head treatment: atosiban vs. terbutaline. |
| 5 | Husslein, P., L. Cabero Roura, J. W. Dudenhausen, H. Helmer, R. Frydman, N. Rizzo and D. Schneider (2007). “Atosiban versus usual care for the management of preterm labor.” J Perinat Med 35(4): 305-313. | atosiban vs. any other tocolytic (other b-agonist, Ca blockers, Mg SO2). |
| 6 | Koks, C. A., H. A. Brolmann, M. J. de Kleine and P. A. Manger (1998). “A randomized comparison of nifedipine and ritodrine for suppression of preterm labor.” European Journal of Obstetrics, Gynecology, & Reproductive Biology 77(2): 171-176. | Head-to-head treatment: ifedipine vs ritodrine. |
| 7 | Lam, F., N. K. Bergauer, S. K. Coleman, G. J. Stanziano and D. Jacques (2000). “A comparison of gestational days gained with oral terbutaline versus continuous subcutaneous terbutaline in women with twin gestations.” Journal of Perinatology 20(7): 408-413 | Head-to-head treatment: oral terbutaline vs subcutaneous terbutaline. |
| 8 | Lam, F., N. K. Bergauer, D. Jacques, S. K. Coleman and G. J. Stanziano (2001). “Clinical and cost-effectiveness of continuous subcutaneous terbutaline versus oral tocolytics for treatment of recurrent preterm labor in twin gestations.” Journal of Perinatology 21(7): 444-450. | Head-to-head treatment: oral terbutaline vs subcutaneous terbutaline. |
| 9 | Rayburn, W., E. Piehl, M. A. Schork and J. Kirscht (1986). “Intravenous ritodrine therapy: a comparison between twin and singleton gestations.” Obstetrics & Gynecology 67(2): 243-248. | Comparison between singleton and twin pregnancies in which both groups were given ritodrine. |
| 10 | Salim, R., G. Garmi, Z. Nachum, N. Zafran, S. Baram and E. Shalev (2012). “Nifedipine compared with atosiban for treating preterm labor: a randomized controlled trial.” Obstetrics & Gynecology 120(6): 1323-1331. | Head-to-head treatment: nifedipine vs. atosiban |
| 12 | al-Najashi, S. S. and A. A. al-Mulhim (1996). "Prolongation of pregnancy in multiple pregnancy." International Journal of Gynaecology & Obstetrics 54(2): 131-135. | Prophylactic trial and no indication for tocolytic treatment for eminent preterm birth.. |
| 13 | O'Connor, M. C., H. Murphy and I. J. Dalrymple (1979). "Double blind trial of ritodrine and placebo in twin pregnancy." British Journal of Obstetrics & Gynaecology 86(9): 706-709. | Prophylactic trial and no indication for tocolytic treatment for eminent preterm birth. |
| 14 | Gummerus, M. and O. Halonen (1987). "Prophylactic long-term oral tocolysis of multiple pregnancies." British Journal of Obstetrics & Gynaecology 94(3): 249-251. | Prophylactic trial and no indication for tocolytic treatment for eminent preterm birth. |
| 15 | Ashworth, M. F., S. F. Spooner, D. A. Verkuyl, R. Waterman and H. M. Ashurst (1990). "Failure to prevent preterm labour and delivery in twin pregnancy using prophylactic oral salbutamol." British Journal of Obstetrics & Gynaecology 97(10): 878-882. | Prophylactic trial and no indication for tocolytic treatment for eminent preterm birth. |
| **c. Growth-restricted fetuses** | | |
| 1 | Cabero, L., M. J. Cerqueira, J. del Solar, J. Bellart and J. Esteban-Altirriba (1988). "Long-term hospitalization and beta-mimetic therapy in the treatment of intrauterine growth retardation of unknown etiology." Journal of Perinatal Medicine 16(5-6): 453-458. | Prophylactic trial and no indication for tocolytic treatment for eminent preterm birth. |
