# Supplementary material for: Tocolysis for inhibiting preterm birth in extremely preterm birth, multiple gestations and in growth-restricted fetuses: a systematic review and meta-analysis
Source: Reprod Health. 2016 Jan 14;13:4. doi: 10.1186/s12978-015-0115-7 (PMC4712490; doi:10.1186/s12978-015-0115-7)

**Additional file 3: Risk of bias within studies**

**1) Risk of bias assessment for randomized controlled trials based on the Cochrane Collaboration’s risk of bias criteria**

| **Extremely preterm birth (RCTs)** | | | |
| --- | --- | --- | --- |
|  | **Study ID** | Richter 2005 | |
|  | **Type of study** | RCT | |
|  | **Bias** | **Author’s judgment** | **Support for judgment** |
|  | Sequence generation | Unclear | Not described |
|  | Allocation concealment | Unclear | Not described |
|  | Blinding of participants and personnel | High | Not blinded |
|  | Blinding of outcomes | Unclear | Not mentioned |
|  | Incomplete outcome data | Low | All patients completed study |
|  | Selective reporting | Low | All specified outcomes in methods reported following the standard protocol |
|  | Other bias | Unclear | Insufficient information to assess other bias exsit |
|  | **Study ID** | Romero 2000 | |
|  | **Type of study** | RCT | |
|  | **Bias** | **Author’s judgment** | **Support for judgment** |
|  | Sequence generation | Low | Computer-generated randomization |
|  | Allocation concealment | Low | Opaque envelopes |
|  | Blinding of participants and personnel | Low | Double blinded |
|  | Blinding of outcomes | Low | Blinded |
|  | Incomplete outcome data | Low | Intended to treat; same numbers, same reasons for attrition |
|  | Selective reporting | Low | Study protocol available and same outcomes reported |
|  | Other bias | Unclear | Imbalance of baseline characteristics, with younger preterm births allocated to atosiban group |
|  | **Study ID** | The Canadian Preterm Labor Investigators Group (PLIG) 1992 | |
|  | **Type of study** | RCT | |
|  | **Bias** | **Author’s judgment** | **Support for judgment** |
|  | Sequence generation | Low | Patients, physicians and nurses blinded to women’s treatment allocation |
|  | Allocation concealment | Low | Done by pharmacy–controlled randomisation |
|  | Blinding of participants and personnel | Low | Double blinded |
|  | Blinding of outcomes | Low | Blinded |
|  | Incomplete outcome data | Unclear | Loss to follow-up unclear, not explained in detail |
|  | Selective reporting | Low | Study protocol available and all expected outcomes reported |
|  | Other bias | Low | The study appears to be free of other sources of bias |

**2) Risk of bias summary for studies included extremely preterm birth (RCTs)**


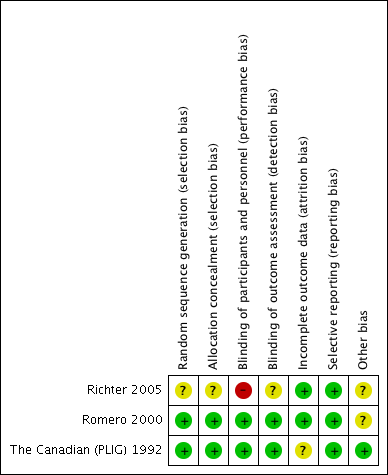

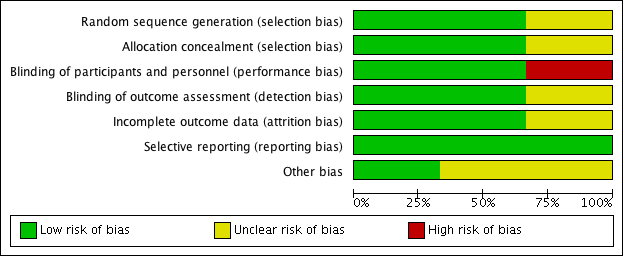


**3) Risk of bias assessment for non-randomized studies based on the Risk of Bias Assessment tool for Non-randomized Studies’ criteria (RoBANS)**

| **Extremely preterm birth (non-RCTs)** | | | |
| --- | --- | --- | --- |
|  | **Study ID** | Berghella 2009 | |
|  | **Type of study** | Retrospective cohorts | |
|  | **Bias** | **Author’s judgment** | **Support for judgment** |
|  | Selection of participants | Low | All women with high risk preterm select from medical database |
|  | Confounding variables | Low | Confounding variables adjusted by multivariable regression |
|  | Measurement of exposure | Low | Medical examination |
|  | Blinding of outcomes | Low | Not blinded, but outcome most likely not influenced |
|  | Incomplete outcome data | Unclear | The number of excluded or missing data were not described |
|  | Selective reporting | Low | Results correspond to intended estimate outcomes reported |
|  | **Study ID** | Cape 2010 | |
|  | **Type of study** | Retrospective cohorts | |
|  | **Bias** | **Author’s judgment** | **Support for judgment** |
|  | Selection of participants | Unclear | Population’s origin not described |
|  | Confounding variables | Low | Adjusted through Cox regression |
|  | Measurement of exposure | Unclear | Although source is unclear, information was only found in medical records |
|  | Blinding of outcomes | Low | Not blinded, but outcome most likely not influenced |
|  | Incomplete outcome data | Unclear | Not mentioned clearly |
|  | Selective reporting | Unclear | Abstract report contained too few information |
|  | **Study ID** | Manuck 2012 | |
|  | **Type of study** | Retrospective cohort | |
|  | **Bias** | **Author’s judgment** | **Support for judgment** |
|  | Selection of participants | Low | All women with high risk preterm select from a single healthcare system database |
|  | Confounding variables | High | Not adjusted |
|  | Measurement of exposure | Low | Medical records |
|  | Blinding of outcomes | Low | Not blinded, but outcome most likely not influenced |
|  | Incomplete outcome data | Unclear | Not mentioned |
|  | Selective reporting | Unclear | Abstract report contained too few information |
|  | **Study ID** | Visintine 2008 | |
|  | **Type of study** | Retrospective cohort | |
|  | **Bias** | **Author’s judgment** | **Support for judgment** |
|  | Selection of participants | Low | All women in the study have same risk and in same University database |
|  | Confounding variables | Low | Confounding variables adjusted by logistic regression |
|  | Measurement of exposure | Low | Medical records |
|  | Blinding of outcomes | Low | Not blinded, but outcome most likely not influenced |
|  | Incomplete outcome data | Low | Identified data and loss to follow-up described |
|  | Selective reporting | High | Side effects and adverse outcomes not assessed |

**4) Risk of bias summary for studies included extremely preterm birth (non-RCTs)**


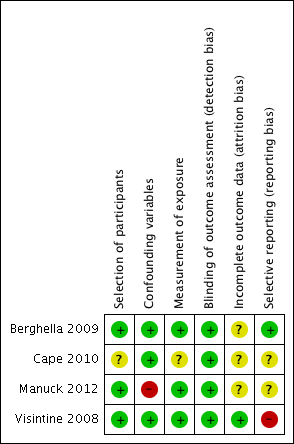

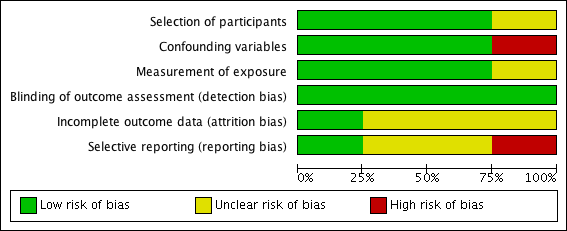

Supplement: Supplementary file 3 — Risk of bias within studies. (DOCX 188 kb) [file 12978_2015_115_MOESM3_ESM.docx]
