# Supplementary material for: Tocolysis for inhibiting preterm birth in extremely preterm birth, multiple gestations and in growth-restricted fetuses: a systematic review and meta-analysis
Source: Reprod Health. 2016 Jan 14;13:4. doi: 10.1186/s12978-015-0115-7 (PMC4712490; doi:10.1186/s12978-015-0115-7)
Supplement: Supplementary file 4 — GRADE tables. (DOCX 134 kb) [file 12978_2015_115_MOESM4_ESM.docx]

**Additional file 4: GRADE tables**

**Extremely preterm birth**

| **Randomized controlled trial (RCTs)** **Date:**2014-04-02 **Question:**Should tocolysis vs placebo or no treatment be used for extreme preterm births? | | | | | | | | | | | |
| --- | --- | --- | --- | --- | --- | --- | --- | --- | --- | --- | --- |
| **Quality assessment** | | | | | | | **№ of patients** | | **Effect** | | **Quality** |
| **№ of studies** | **Study design** | **Risk of bias** | **Inconsistency** | **Indirectness** | **Imprecision** | **Other considerations** | **Tocolysis** | **Placebo** | **Relative (95% CI)** | **Absolute (95% CI)** |  |
| Prolongation of pregnancy > 24 hours | | | | | | | | | | | |
| 1 | randomised trials | not serious | not serious | not serious | serious ^1^ | none | 29/43 (67.4%) | 20/34 (58.8%) | **RR 1.15** (0.81 to 1.63) | 9 more per 100 (from 11 fewer to 37 more) | ⨁⨁⨁ MODERATE |
| Prolongation of pregnancy > 48 hours | | | | | | | | | | | |
| 2 | randomised trials | serious ^2^ | not serious | not serious | very serious ^1^ | none | 44/63 (69.8%) | 37/54 (68.5%) | **RR 1.40** (0.83 to 1.31) | 27 more per 100 (from 12 fewer to 21 more) | ⨁ VERY LOW |
| Prolongation of pregancy > 7days | | | | | | | | | | | |
| 2 | randomised trials | serious ^2^ | not serious | not serious | very serious ^1^ | none | 40/63 (63.5%) | 35/54 (64.8%) | **RR 1.05** (0.75 to 1.48) | 3 more per 100 (from 16 fewer to 31 more) | ⨁ VERY LOW |
| Perinatal death | | | | | | | | | | | |
| 2 | randomised trials | serious ^3^ | serious ^4^ | not serious | very serious ^5^ | none | 28/134 (20.9%) | 22/131 (16.8%) | **RR 2.22** (0.26 to 19.24) | 20 more per 100 (from 12 fewer to 100 more) | ⨁ VERY LOW |

MD – mean difference, RR – relative risk

1. Total number of cases less than 300
2. Allocation concealment not performed
3. One study with unclear randomization and one study without allocation concealment
4. Large heterogeneity (>60%)
5. Small sample size (<300) and wide confidence interval

| **Non-randomized studies (non-RCTs)**  **Date:**2014-04-02 **Question:**Should tocolysis vs placebo or no treatment be used for extreme preterm births? | | | | | | | | | | | |
| --- | --- | --- | --- | --- | --- | --- | --- | --- | --- | --- | --- |
| **Quality assessment** | | | | | | | **№ of patients** | | **Effect** | | **Quality** |
| **№ of studies** | **Study design** | **Risk of bias** | **Inconsistency** | **Indirectness** | **Imprecision** | **Other considerations** | **Tocolysis** | **No treatment** | **Relative (95% CI)** | **Absolute (95% CI)** |  |
| Prolongation of pregnancy > 7 days | | | | | | | | | | | |
| 1 | non-RCTs | very serious ^1^ | not serious | not serious | serious ^2^ | plausible residual confounding would suggest spurious effect | 28/84 (33.3%) | 10/64 (15.6%) | **RR 2.13** (1.12 to 4.06) | 18 more per 100 (from 2 more to 48 more) | ⨁ VERY LOW |
|  |  |  |  |  |  |  |  | 15.6% |  | 18 more per 100 (from 2 more to 48 more) |  |
| Prolong of pregnancy > 24 weeks | | | | | | | | | | | |
| 1 | non-RCTs | serious ^3^ | not serious | not serious | not serious | none | 40/51 (78.4%) | 43/50 (86.0%) | **RR 0.91** (0.76 to 1.09) | 8 fewer per 100 (from 8 more to 21 fewer) | ⨁ VERY LOW |
|  |  |  |  |  |  |  |  | 86.0% |  | 8 fewer per 100 (from 8 more to 21 fewer) |  |
| Prolongation of pregnancy > 28 weeks | | | | | | | | | | | |
| 1 | non-RCTs | serious ^4^ | not serious | not serious | not serious | none | 34/68 (50.0%) | 85/154 (55.2%) | **RR 0.91** (0.69 to 1.20) | 5 fewer per 100 (from 11 more to 17 fewer) | ⨁ VERY LOW |
|  |  |  |  |  |  |  |  | 55.2% |  | 5 fewer per 100 (from 11 more to 17 fewer) |  |
| Prolongation of pregnancy > 32 weeks | | | | | | | | | | | |
| 2 | non-RCTs | serious ^5^ | not serious | not serious | not serious | none | 63/119 (52.9%) | 105/204 (51.5%) | **RR 0.94** (0.76 to 1.17) | 3 fewer per 100 (from 9 more to 12 fewer) | ⨁ VERY LOW |
|  |  |  |  |  |  |  |  | 57.7% |  | 3 fewer per 100 (from 10 more to 14 fewer) |  |
| Prolongation of pregnancy > 35 weeks | | | | | | | | | | | |
| 2 | non-RCTs | serious ^5^ | not serious | not serious | not serious | none | 55/119 (46.2%) | 87/204 (42.6%) | **RR 0.96** (0.75 to 1.23) | 2 fewer per 100 (from 10 more to 11 fewer) | ⨁ VERY LOW |
|  |  |  |  |  |  |  |  | 50.5% |  | 2 fewer per 100 (from 12 more to 13 fewer) |  |
| Neonatal survival | | | | | | | | | | | |
| 1 | non-RCTs | serious ^4^ | not serious | not serious | not serious | none | 47/68 (69.1%) | 95/154 (61.7%) | **RR 1.12** (0.92 to 1.37) | 7 more per 100 (from 5 fewer to 23 more) | ⨁ VERY LOW |
|  |  |  |  |  |  |  |  | 61.7% |  | 7 more per 100 (from 5 fewer to 23 more) |  |
| Perinatal death | | | | | | | | | | | |
| 2 | non-RCTs | serious ^6^ | not serious | not serious | serious ^2^ | Plausible residual confounding would suggest spurious effect | 51/152 (33.6%) | 94/218 (43.1%) | **RR 0.73** (0.55 to 0.95) | 12 fewer per 100 (from 2 fewer to 19 fewer) | ⨁ VERY LOW |
|  |  |  |  |  |  |  |  | 46.5% |  | 13 fewer per 100 (from 2 fewer to 21 fewer) |  |
| Birth weight >1500 grams | | | | | | | | | | | |
| 1 | non-RCTs | serious ^4^ | not serious | not serious | not serious | none | 30/68 (44.1%) | 76/154 (49.4%) | **RR 0.89** (0.65 to 1.22) | 5 fewer per 100 (from 11 more to 17 fewer) | ⨁ VERY LOW |
|  |  |  |  |  |  |  |  | 49.4% |  | 5 fewer per 100 (from 11 more to 17 fewer) |  |
| Intraventricular hemorrhage | | | | | | | | | | | |
| 1 | non-RCTs | very serious ^7^ | not serious | not serious | not serious | none | 31/69 (44.9%) | 34/69 (49.3%) | **RR 0.91** (0.64 to 1.30) | 4 fewer per 100 (from 15 more to 18 fewer) | ⨁ VERY LOW |
|  |  |  |  |  |  |  |  | 49.3% |  | 4 fewer per 100 (from 15 more to 18 fewer) |  |
| Necrotizing enterocolitis | | | | | | | | | | | |
| 1 | non-RCTs | very serious ^7^ | not serious | not serious | not serious | none | 13/69 (18.8%) | 13/69 (18.8%) | **RR 1.0** (0.5 to 2.0) | 0 fewer per 100 (from 9 fewer to 19 more) | ⨁ VERY LOW |
|  |  |  |  |  |  |  |  | 18.8% |  | 0 fewer per 100 (from 9 fewer to 19 more) |  |
| Patent ductus arteriosus | | | | | | | | | | | |
| 1 | non-RCTs | very serious ^7^ | not serious | not serious | not serious | none | 58/69 (84.1%) | 50/69 (72.5%) | **RR 1.16** (0.97 to 1.39) | 12 more per 100 (from 2 fewer to 28 more) | ⨁ VERY LOW |
|  |  |  |  |  |  |  |  | 72.5% |  | 12 more per 100 (from 2 fewer to 28 more) |  |
| Spontaneous intestinal perforations | | | | | | | | | | | |
| 1 | non-RCTs | very serious ^7^ | not serious | not serious | not serious | none | 8/69 (11.6%) | 3/69 (4.3%) | **RR 2.67** (0.74 to 9.63) | 7 more per 100 (from 1 fewer to 38 more) | ⨁ VERY LOW |
|  |  |  |  |  |  |  |  | 4.3% |  | 7 more per 100 (from 1 fewer to 38 more) |  |

MD – mean difference, RR – relative risk

1. The information was from a study with high risk of confounding variables and unclear risk of outcome data reporting
2. A wide confidence interval without confounding variable adjusted
3. The information was from a study with high risk of selective reporting
4. The information was from a study with unclear risk of incomplete outcome data
5. The information was from two studies with unclear risk of incomplete outcome data in one study and high risk of selective reporting in another study
6. This information was from two studies with unclear risk of incomplete outcome data and on study with high risk of cofounding variables and unclear selective reporting
7. This information was from a study with unclear risk of selection of participants, measurement of exposure, incomplete data and selective reporting
